# Association of CD99 short and long forms with MHC class I, MHC class II and tetraspanin CD81 and recruitment into immunological synapses

**DOI:** 10.1186/1756-0500-4-293

**Published:** 2011-08-13

**Authors:** Supansa Pata, Pavel Otáhal, Tomáš Brdička, Witida Laopajon, Kodchakorn Mahasongkram, Watchara Kasinrerk

**Affiliations:** 1 Division of Clinical Immunology, Department of Medical Technology, Faculty of Associated Medical Sciences, Chiang Mai University, Chiang Mai 50200, Thailand; 2 Laboratory of Molecular Immunology, Institute of Molecular Genetics AS CR, Prague 14220, Czech Republic; 3 Biomedical Technology Research Center, National Center for Genetic Engineering and Biotechnology, National Science and Technology Development Agency at the Faculty of Associated Medical Sciences, Chiang Mai University, Chiang Mai 50200, Thailand

## Abstract

**Background:**

CD99, a leukocyte surface glycoprotein, is broadly expressed in many cell types. On the cell surface, CD99 is expressed as two distinct isoforms, a long form and a short form. CD99 has been demonstrated to play a key role in several biological processes, including the regulation of T cell activation. However, the molecular mechanisms by which CD99 participates in such processes are unclear. As CD99 contains a short cytoplasmic tail, it is unlikely that CD99 itself takes part in its multi-functions. Association of CD99 with other membrane proteins has been suggested to be necessary for exerting its functions.

**Results:**

In this study, we analyzed the association of CD99 with other cell surface molecules involved in T cell activation. We demonstrate the association of MHC class I, MHC class II and tetraspanin CD81 with CD99 molecules on the cell surface. Association of CD99 with its partners was observed for both isoforms. In addition, we determined that CD99 is a lipid raft-associated membrane protein and is recruited into the immunologic synapse during T cell activation. The implication of CD99 on T cell activation was investigated. Inhibition of anti-CD3 induced T cell proliferation by an anti-CD99 monoclonal antibody was observed.

**Conclusions:**

We provide evidence that CD99 directly interact and form the complex with the MHC class I and II, and tetraspanin CD81, and is functionally linked to the formation of the immunologic synapse. Upon T cell activation, CD99 engagement can inhibit T cell proliferation. We speculate that the CD99-MHC-CD81 complex is a tetraspanin web that plays an important role in T cell activation.

## Background

Upon T cell activation, T cell stimulation is initiated when a T cell receptor (TCR) encounters specific antigen peptide-MHC complexes expressed on the surface of antigen presenting cells (APCs). The interaction of various co-stimulatory molecules expressed on T cells and APCs is, in addition, involved in the induction of proper T cell responses. These interactions induce the formation of an immunological synapse (IS) at the cell-cell junction between T cells and APCs, resulting in the reorganization of the related cell membrane signaling molecules in a concerted fashion [1,2]. The IS is proposed to function as a platform for signal transduction and cytoskeleton reorganization, which is essential for the determination of TCR sensitivity and responsiveness. Several co-stimulatory molecules have been shown to translocate into the IS and are crucial in determining antigen-specific T cell activation and tolerance [2,3]. CD99 has been recently demonstrated to function as a co-stimulatory molecule in T cell activation [4]. Co-ligation of CD99 and CD3 molecules leads to the translocation of TCR complexes into the IS and enhances TCR signaling events.

CD99 is a type 1 transmembrane glycoprotein encoded by the MIC2 gene, and shares no significant homology with any known protein family [5-9]. The CD99 molecule contains an extracellular domain, followed by a transmembrane domain and a short 36-amino acid intracytoplasmic domain [9]. CD99 is broadly distributed among many cell types, both hematopoietic and non-hematopoietic cells [10-14]. Although the functional role of CD99 is not yet fully understood, it has been implicated in multiple cellular events. CD99 has been described as a T-cell co-stimulator and regulator of cytokine production [4,15]. Engagement of CD99 with agonistic antibodies induced apoptosis of immune cells and tumor cells [14,16,17]. CD99 ligation was also demonstrated to induce expression of adhesion molecules, including ELAM-1, VCAM-1 and ICAM-1, which are associated with leukocyte adhesion and transendothelial migration [13,14,18-24]. Furthermore, CD99 engagement has been reported to induce the expression of TCR, MHC class I and MHC class II by accelerated mobilization of these molecules from the Golgi compartment to the plasma membrane [25]. Requirement of CD99 expression in IFN-γ induced MHC class I expression has also been observed [26]. Without CD99, upon IFN-γ stimulation, MHC class I molecules became accumulated within the Golgi apparatus [26].

Signaling pathways triggered by CD99 have been elucidated in several studies. Stimulation of CD99 with agonistic antibodies enhanced the expression of several T cell activation markers on anti-CD3-activating T cells, elevation of intracellular Ca2^+ ^and the tyrosine phosphorylation of cellular proteins [15,27]. We have demonstrated that protein kinase C inhibitor, sphingosine and a protein tyrosine kinase inhibitor, genistein, blocked cell aggregation induced by CD99 engagement [13]. It has also been reported that CD99 ligation induced differential activation of three mitogen-activated protein kinase (MAPK) members, ERK, JNK and p38 MAPK [28]. Activation of src kinase and focal adhesion kinase (FAK) by CD99 molecules has also been demonstrated [29]. Although several lines of evidence indicate the involvement of CD99 in cell signaling, with its short cytoplasmic tail, it is unlikely that CD99 itself takes part in signaling events. In the cellular context, association of CD99 with other membrane proteins has been suggested to be necessary for exerting its functions.

On the cell surface, CD99 is expressed as two distinct isoforms depending on the alternative splicing of the encoding gene [22]. The long form (type I) contains 185 amino acid residues and its mobility in SDS-PAGE corresponds to an apparent molecular weight (MW) of 32 kDa. The short form (Type II; 161 residues, apparent MW of 28 kDa) harbors a deletion in the cytoplasmic segment. The CD99 isoforms are differentially expressed in a cell type-specific manner among hematopoietic cells and cell lines [16,22]. The CD99 isoform expression was shown to dictate distinct functional events [27,30,31]. Expression of the long form in CD99-deficient Jurkat T cell line is sufficient to promote CD99-induced cell adhesion, whereas co-expression of the two isoforms is required to trigger T cell death [16]. In addition, on B cells, the short form of CD99 inhibited homotypic adhesion, while the activation of the CD99 long form promoted cell-cell adhesion. The opposing effects of CD99 isoforms on homotypic B cell aggregation were shown to be due to their opposing functions in controlling the expression of the cell adhesion molecule, LFA-1 [22].

Although the CD99 molecule has been described as a multi-functional cell surface molecule, it contains a short intracellular domain [9]. Interaction with other cell surface molecules is, therefore, assumed to be necessary in order to regulate its multiple functions. In this study, we explored the possibility that CD99 may form a microdomain with other proteins on the cell surface. We demonstrate here the association of MHC class I, MHC class II and a tetraspanin CD81, with both CD99 isoforms. Upon T cell activation, translocation of CD99 into IS and inhibition of T cell proliferation by anti-CD99 monoclonal antibody (mAb) were observed, indicating an important role for CD99 in T cell activation.

## Results

### Association of CD99 with various membrane proteins

**Figure 1:**
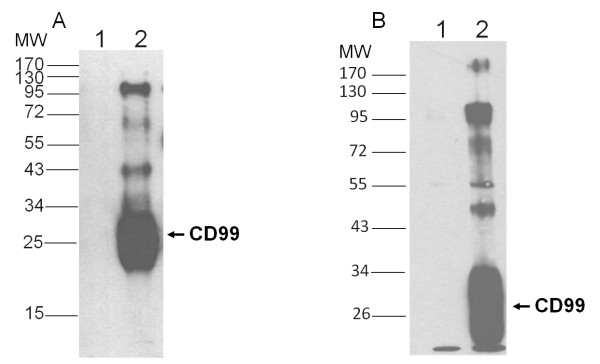
**Co-immunoprecipitation of CD99 with other membrane proteins**. Immunoprecipitation of biotinylated Jurkat cell lysates using mild detergent 1% Brij-58 (A) or raft-disrupting detergent 1% Lauryl maltoside (B). Cell lysates were precipitated using isotype matched control 4G2 mAb (lane 1) and anti-CD99 mAb MT99/3 (lane 2). Electrophoresis was performed under reducing conditions. The positions of molecular mass markers are indicated on the left in kDa. The positions of CD99 molecules are indicated.

To investigate whether CD99 was associated with other proteins on the cell surface, co-immunoprecipitations of biotinylated Jurkat cell lysates using anti-CD99 mAb MT99/3 [13] were performed. As shown in Figure 1A, using 1% nonionic detergent Brij-58, a number of membrane proteins with molecular weights of 110, 50 and 40 kDa were co-precipitated with the CD99 molecules. To exclude the possibility that the observed co-precipitation of CD99 with other proteins is due to the artificial effect of lipid raft association, co-immunoprecipitation using the lipid raft disrupting detergent 1% LM was carried out. Similar results were obtained (Figure 1B) indicating that, at the cell surface, CD99 is associated with other membrane molecules.

### Association of CD99 with MHC class I and MHC class II

**Figure 2:**
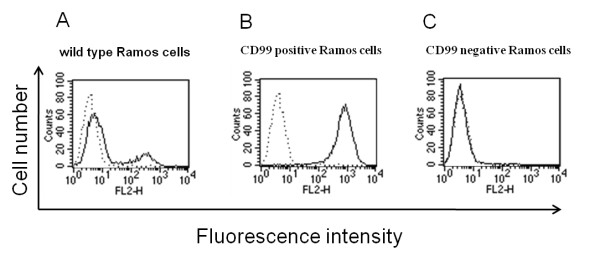
**Immunofluorescenence analysis of CD99 expression on Ramos wild type and CD99 sorted cells**. Ramos cells were chosen for establishment of CD99 expressing and non-expressing cells by an immunomagnetic sorting system using anti-CD99 mAb. The sorted cells were then verified for their surface CD99 expression by immunoflurorescence and flow cytometry. The wild type (A), CD99 positive (B) and CD99 negative (C) cells were stained with anti-CD99 mAbs MT99/3 and PE-conjugated anti-mouse immunoglobulin antibodies. Solid lines represent the immunofluorescence profiles of the cells stained with anti-CD99 mAb and dashed lines represent background fluorescence of conjugate control.

As CD99 has been reported to transport MHC class I and class II to the cell surface [25,32], we speculated that CD99 molecules may be associated with the transported molecules. To verify this hypothesis, immunoprecipitation using anti-CD99 mAb was carried out. The immunoprecipitated proteins were then analyzed by Western immunoblotting using the anti-MHC class I and class II mAbs. However, to obtain more precise results, we first established CD99 expressing and CD99 non-expressing cells. In order to obtain the appropriate cell type for preparing CD99 expressing and non-expressing cells, several cell lines were stained with anti-CD99 mAb and analyzed by flow cytometry. It was found that all of the tested cell lines, including Ramos, Raji, Jurkat, Molt4, U937 and THP-1, were positive with anti-CD99 mAb (data not shown). However, among the tested cell lines, only Ramos (a human Burkitt's lymphoma cell line) expressed CD99 in a heterogeneous manner, i.e., there were CD99 expressing and non-expressing populations (Figure 2A). The heterogeneous Ramos cells were then selected and separated by immunomagnetic sorting resulting in CD99 positive and CD99 negative populations (Figure 2B and 2C). During long-term cultivation, the CD99 negative cells retained their CD99 negative phenotype, while the CD99 positive population returned to the wild type heterogeneous profile (data not shown). Hence, the Ramos wild type and CD99 negative cells were employed for further co-immunoprecipitation experiments.

**Figure 3:**
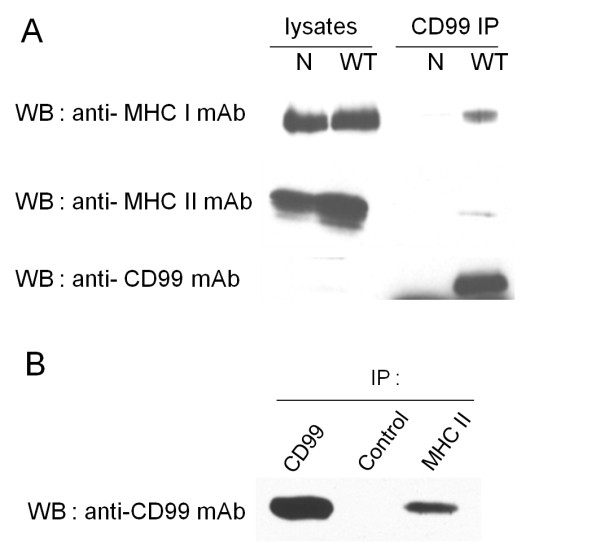
**Interaction between CD99 and MHC class I and MHC class II**. (A) Cell lysates (lysate) or CD99 immunoprecipitated proteins (CD99 IP) of wild type (WT) or CD99 negative (N) Ramos cells were analyzed by Western immunoblotting using the indicated mAbs. (B) Wild type Raji cell lysates were immunoprecipitated using anti-CD99 (CD99), anti-MHC class II (MHC II) and isotype matched control mAbs (control) and analyzed by Western immunoblotting by using anti-CD99 mAb MT99/3.

As shown in Figure 3A, when using wild type Ramos cells, MHC class I and MHC class II were co-precipitated with the CD99 molecules. The MHC class II, however, was demonstrated to co-precipitate with CD99 in lower amount than the MHC class I molecules (Figure 3A). In contrast, no MHC class I or class II were detected in the immunoprecipitates resulting from CD99 negative Ramos cells. CD99, as expected, was detected only in immunoprecipitates of the wild type, but not of the CD99 negative Ramos cells. These findings indicated that CD99 physically interacted with MHC class I and class II. Western immunoblotting of cell lysates prior to immunoprecipitation were also performed. MHC class I and class II were present in similar amounts in both the wild type and CD99 negative Ramos cell lysates (Figure 3A), indicating these molecules can be normally expressed even in the absence of CD99. In the presence of CD99, nevertheless, the MHC molecules were expressed as CD99-MHC complexes. We note that, in the Western immunoblotting experiment using anti-CD99 mAb, CD99 could not be detected in either wild type or CD99 negative Ramos cell lysates (Figure 3A). It is likely that, as the wild type Ramos cells expressed CD99 in a heterogeneous pattern in which a small CD99 positive population was present (Figure 2A), a small amount of CD99 molecules are contained in the whole cell lysates. For this reason, the CD99 molecule could not be detected by Western immunoblotting.

To confirm the observed association of CD99 with MHC molecules, reciprocal co-immunoprecipitation experiments were carried out. In accordance with the above results, CD99 was observed in the immunoprecipitates using anti-MHC class II mAb (Figure 3B). However, CD99 could not be detected in the immunoprecipitates using anti-MHC class I mAb (data not shown). This may be because of the low affinity of the employed anti-MHC class I mAb itself, resulting in it being inappropriate for immunoprecipitation. Taken together, our findings suggest that CD99 form complexes with MHC class I and MHC class II molecules.

### Association of CD99-MHC complexes with tetraspanin CD81

**Figure 4:**
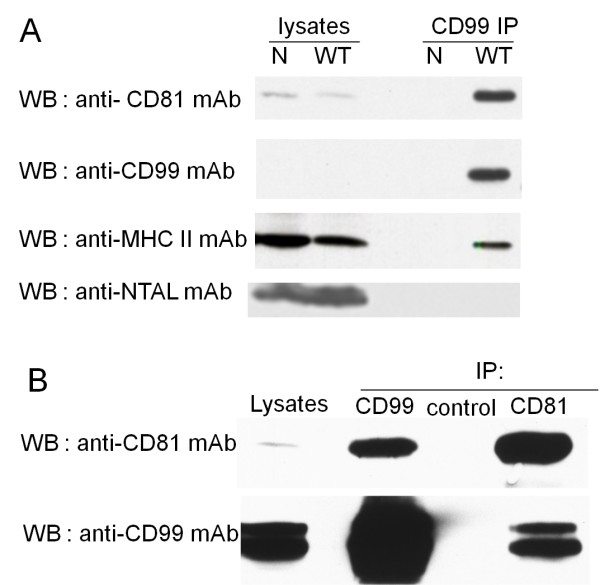
**Association of CD99-MHC class II complexes with CD81**. (A) Cell lysates (lysate) or CD99 immunoprecipitated proteins (CD99 IP) of wild type (WT) or CD99 negative (N) Ramos cells were analyzed by Western immunoblotting using the indicated mAbs. (B) Jurkat cell lysates were immunoprecipitated with the anti-CD99 (CD99), anti-CD81 (CD81) or isotype matched control mAb (control) and analyzed by Western immunoblotting using the indicated mAbs. The cell lysate (Lysate) prior to immunoprecipitation was also analyzed by Western immunoblotting using the indicated mAbs.

Tetraspanins are broadly expressed cell surface proteins that span the cell membrane four times [33]. All cells of the immune system express tetraspanins [34], which provide a scaffold that facilitates the spatial and temporal engagement of their associated proteins [34,35]. The tetraspanins have been implicated in several cellular functions such as cell growth, differentiation, intercellular adhesion, motility, and intracellular signaling [33,35]. Associations of tetraspanins with MHC class I and class II have been previously described [33,35-37]. CD81, a member of the tetraspanins, is expressed on T and B lymphocytes and functions as a co-stimulatory molecule. The associations of CD81 with MHC molecules have been described [36,37]. Following our finding of an association between the MHC and CD99 molecules, we investigated whether the CD99-MHC complex also contained tetraspanins, CD81. Immunoprecipitated proteins of wild type and CD99 negative Ramos cells were analyzed by Western immunoblotting using mAb against a tetraspanin, CD81. CD81, as well as MHC class II and CD99, were detected in the CD99 immunoprecipitates of the wild type, but not the CD99 negative Ramos cells (Figure 4A). To exclude any possible artificial effect of lipid raft association, we verified the presence of a typical lipid raft containing molecule, NTAL [38], in the CD99 immunoprecipitates. As expected, NTAL was not found in the CD99 immunoprecipitates (Figure 4A) indicating that the association observed is not due to the occurrence of lipid raft association. Correspondingly, using Jurkat cells, CD81 and CD99 were observed in the CD99 immunoprecipitates (Figure 4B), but not in that using an irrelevant control mAb. Concordantly, the reciprocal co-immunoprecipitation showed that CD99 and CD81 were detected in the CD81 immunoprecipitates (Figure 4B). As Jurkat cells express both CD99 long and short forms, Western immunoblotting obtained by using anti-CD99 mAb demonstrated 2 bands corresponding to the 32 kDa long form and the 28 kDa short form. Collectively, our results indicate that tetraspanin CD81 is associated with the CD99-MHC complexes.

### Colocalization of CD99 with MHC class I, MHC class II and tetraspanin CD81

**Figure 5:**
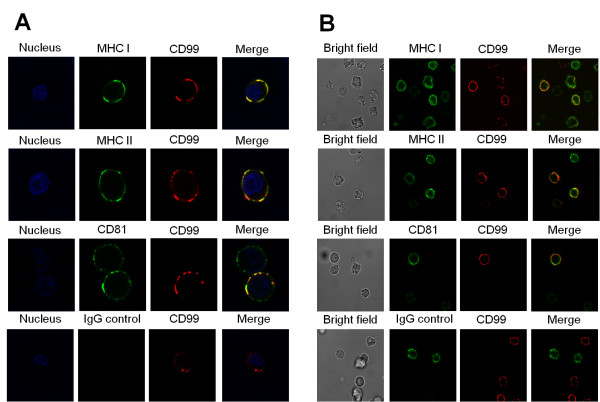
**Colocalization of CD99 and MHC class I, MHC class II and CD81**. Raji cells (A) or PBMCs (B) were stained with anti-MHC class I (MHC I), anti-MHC class II (MHC II), anti-CD81 (CD81) and anti-CD99 (CD99) mAbs as described in Materials and Methods. In IgG control, PB1 mAb (for Raji) or MEM-111 mAb (for PBMCs) were used instead of anti-MHC or anti-CD81 mAb. The stained cells were analyzed for colocailization by a confocal microscope. The green images represent the distribution of MHC class I, MHC class II, CD81 molecules and red images represent the CD99 molecules. Cell nuclei are shown in blue.

To further elucidate the association of CD99 with MHC and CD81 molecules, colocalization of CD99 and MHC class I, MHC class II or CD81 were visualized by laser confocal microscopy. Colocalizations of CD99 and MHC class I, CD99 and MHC class II as well as CD99 and CD81 were demonstrated in Raji cell line (Figure 5A). We note that, in the CD99 and CD81 panel (Figure 5A), a CD99 negative/CD81 positive cell was captured. This cell expressed only the green fluorescence of CD81 in all confocal images, indicating no cross-reactivity between conjugates and the primary antibodies used. It is important to mention that not all of the CD99 molecules and MHC or CD81 were associated; some molecules were expressed in an un-associated form. The confocal imaging results, consistent with the co-immunoprecipitation experiments, strongly suggest that CD99 is associated with MHC class I, MHC class II and CD81 on the cell surface.

To demonstrate whether the association of CD99 molecules with its interacting partners is also observed in peripheral blood cells, colocalization of CD99 with MHC and CD81 molecules on PBMCs' membrane were determined by confocal microscopic analysis. As shown in Figure 5B, colocalization of CD99-MHC class I, CD99-MHC class II and CD99-CD81 were observed. The colocalizations were observed only in cells expressing CD99 and MHC or CD81. In IgG control, anti-CD54 mAb was used instead of anti-MHC class I, class II or CD81 and found no colocalization between the CD99 and CD54 molecules indicating no cross-reactivity between conjugates and the primary antibodies used. These results indicated that association of CD99 with MHC and CD81 is physically occurred in peripheral blood cells.

### Association of CD99 short and long forms with MHC class I, MHC class II and CD81

**Figure 6:**
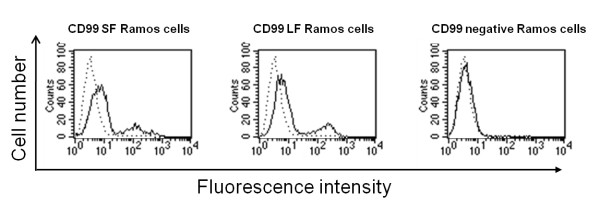
**Flow cytometric analysis of CD99 expression of Ramos expressing CD99 short and long forms**. CD99 negative Ramos cells were transduced with retrovirus carrying CD99 short form (CD99 SF Ramos cells) or CD99 long form (CD99 LF Ramos cells). The transduced cells were stained with the anti-CD99 mAb MT99/3 and counterstained with PE-conjugated anti-mouse immunoglobulins antibodies. Solid lines represent the immunofluorescence profiles of the cells stained with anti-CD99 mAb and dashed lines represent background fluorescence of conjugate control.

**Figure 7:**
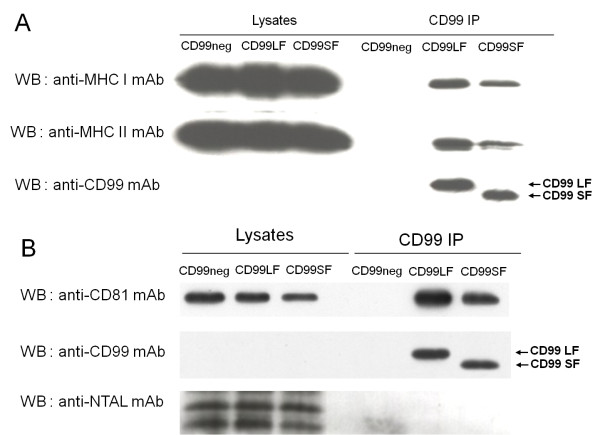
**Interaction between CD99 isoforms with MHC class I, class II and CD81**. Cell lysates (Lysate) or CD99 immunoprecipitates (CD99 IP) of Ramos expressing CD99 short form (CD99SF), CD99 long form (CD99LF) and CD99 negative Ramos (CD99neg) were analyzed by Western immunoblotting using anti-MHC class I, anti-MHC class II and anti-CD99 mAbs (A) or anti-CD81, anti-CD99 and anti-NTAL mAbs (B). The positions of CD99 short and long isoforms are indicated.

CD99 molecules are expressed on the cell surface in two isoforms, a 28 kDa short form and 32 kDa long form. As described above, we have demonstrated that MHC class I, MHC class II, and CD81 are associated with the CD99 molecules. To determine whether this association is dependent on the CD99 isoforms, we have developed Ramos cells expressing CD99 short and long forms, using retroviral vectors encoding the respective isoforms. The expression of CD99 in the generated stable cell lines was verified by flow cytometry (Figure 6A) and immunoprecipitation (Figure 7). Immunoprecipitation of CD99 molecules followed by Western immunoblotting with anti-CD99 mAb demonstrated the expected surface expression patterns on the transduced cells (Figure 7).

We performed immunoprecipitations from the lysates of cell lines expressing selectively the two CD99 isoforms using anti-CD99 mAb and analyzed the immunoprecipitates by Western immunoblotting. As shown in Figure 7A and 7B, MHC class I, MHC class II, and CD81 could be detected in the immunoprecipitates obtained from cells expressing both the CD99 short and long forms. No immunoreactive band could be observed in the control immunoprecipitate of CD99 negative Ramos cell lysates. As anticipated, the typical lipid raft containing molecule NTAL was not detected in the CD99 immunoprecipitated proteins (Figure 7B). Collectively, the results suggest that the association of MHC class I, MHC class II and CD81 with CD99 can be observed in both CD99 isoforms.

### Recruitment of CD99 into the immunological synapse (IS)

**Figure 8:**
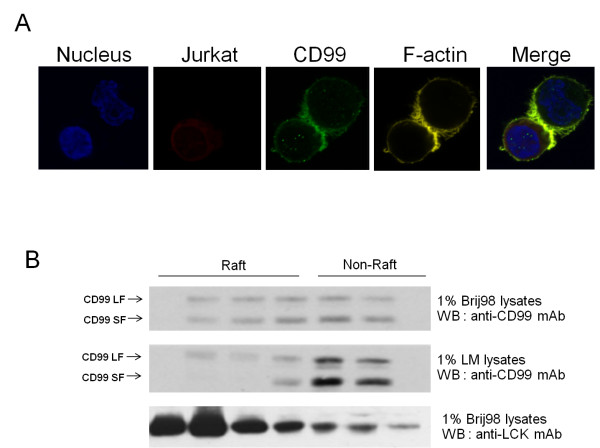
**CD99 is recruited into the IS and lipid rafts**. (A) DDAO-SE pre-labeled Jurkat T cells were incubated with SEB-loaded Raji B cells. Cells were then plated onto a cover slip, fixed, stained with anti-CD99 mAb followed by Alexa Fluor 488-conjugated goat anti-mouse IgG antibodies, TRITC-phalloidin for F-actin and Hoechst 33258 for the nucleus. The stained cells were visualized by confocal fluorescence microscopy. The green and yellow images represent the localization and distribution of CD99 and F-actin, respectively. The nuclei and Jurkat cells are shown in blue and red, respectively. (B) Jurkat cells were homogenized to isolate cell membranes, which were then solubilized in lipid raft preserving detergent 1% Brij-98 or lipid raft disrupting detergent 1% Lauryl maltoside (LM) and subjected to gel filtration on Sepharose 4B. The obtained fractions were analyzed by Western immunoblotting using anti-CD99 and anti-Lck mAbs.

Since our results suggest that MHC class I, class II and CD81, which are accessory molecules for T cell activation and are accumulated in the IS, are associated with CD99, we further investigated whether CD99 also appears at the IS during antigen presentation. We loaded Raji B cells with SEB and incubated them with DDAO-SE pre-labeled Jurkat T cells. As shown in Figure 8A, CD99 accumulated at the T-B cell interface. Colocalization of CD99 with F-actin, a marker of IS formation, was also observed. These data suggest that CD99 is recruited to the IS.

Immune cell signaling pathways are at least partially mediated by lipid raft-associated proteins. During T cell activation, lipid rafts are accumulated in the IS and function as the platform for signal molecules [39]. We thus attempted to confirm whether, in the Jurkat T cell line, CD99 is a lipid raft-associated membrane protein. As shown in Figure 8B, in the presence of mild lipid raft-preserving detergent 1% Brij-98, both short and long forms of CD99 were found in the raft and non-raft fractions. In contrast, in the presence of the raft disrupting detergent 1% LM, CD99 was mostly found in the non-raft fractions. The lipid raft containing protein Lck was included in the experiment as a positive control and was observed in the lipid raft fractions. These results indicate that CD99 molecules are recruited to the IS and a fraction of CD99 is associated with lipid rafts.

### Inhibition of T cell proliferation by engagement of surface CD99

**Figure 9:**
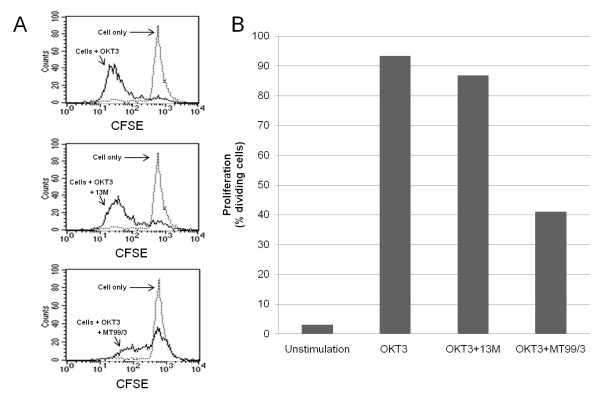
**Engagement of CD99 inhibits T cell proliferation**. PBMCs were labeled with CFSE and cultured with or without anti-CD3 mAb OKT3 in the absence or presence of anti-CD99 mAb MT99/3 or isotype matched control mAb 13 M for 5 days. Cells were then harvested and acquired in a flow cytometer. (A) FACS profiles illustrate the T-cell proliferation levels obtained in the different indicated conditions, as determined by CFSE fluorescence loss. (B) Histogram plots show the level of cell proliferation as determined by the percentage of dividing cells in the indicated culture conditions.

To determine whether CD99 is involved in T cell activation, peripheral blood mononuclear cells (PBMCs) were activated with anti-CD3 mAb OKT3 in the presence of anti-CD99 mAb MT99/3 and cell proliferation was verified. As shown in Figure 9, engagement of CD99 inhibited T cell proliferation. In contrast, the isotype matched control mAb did not show any inhibitory effect. These results indicate that CD99 play a regulatory role in T cell activation.

## Discussion

Cell surface molecules containing a short cytoplasmic tail usually form complexes with other molecules in order to modulate their signal transduction and functions. Identification of the interacting partners of cell surface molecules may lead to a better understanding of cellular function and immune responses. In the present study, we have identified several interacting partners of a multi-functional membrane protein, CD99. We observed that on the cell surface, CD99 molecules are associated with MHC class I, MHC class II and tetraspanin CD81. The CD99 molecule has been described by several studies as a signaling molecule. Engagement of CD99 induces signal transduction, resulting in the regulation of T cell activation, cell adhesion, cell migration, and cell death. However, CD99 itself contains a short intracellular domain without signaling motifs on the cytoplasmic tail except a site for PKCα phosphorylation [9,30]. Signal transduction induced by CD99 is, therefore, incompletely understood. Since we found, in this study, that CD99 form complexes with other signal mediated molecules, i.e., MHC class I, MHC class II and tetraspanin CD81, this may help to clarify the precise signaling mechanism of the CD99 molecule and provide better understanding of its functional roles.

MHC molecules are cell surface proteins that play a critical role in antigen presentation. CD99 has been reported to regulate the expression of MHC class I and class II [25,26,32]. Up-regulation of MHC class I and class II by CD99 results from accelerated intracytoplasmic transportation of MHC molecules to the plasma membrane rather than de novo synthesis of these molecules [25,32]. Deceased expression of CD99 resulted in the retention of MHC class I molecules in the Golgi compartment by affecting the transportation of the MHC molecules in the *trans*-Golgi network [26,32]. Moreover, colocalization of CD99 and MHC class I molecules is clearly demonstrated both in the Golgi apparatus and at the cell surface [26]. Strikingly, it was demonstrated that the CD99 and MHC class I association occurs at the transmenbrane domain. Valines located in the transmembrane region of CD99 are required for the binding to MHC molecules, likely in relation with their hydrophobicity [26]. In agreement with the previous reports, in this study, we have shown that, after transportation, the CD99 molecules form complexes with their transported MHC molecules and are co-expressed as microdomains on the cell surface.

In addition to the MHC molecules, we demonstrated the association of CD99 with a tetraspanin, CD81. The tetraspanins are cell surface proteins that are broadly expressed in many cell types. Data from biochemical studies or knockout mice suggest that the tetraspanins play a major role in membrane biology [33,35]. One of the most striking features of tetraspanins is their ability to form a network of multi-molecular complexes, known as the tetraspanin web, between each individual tetraspanin and other surface proteins. Within the immune cells, all cells express tetraspanins, which provide a scaffold that facilitates the spatial and temporal engagement of their associated proteins. Tetraspanins and their associated proteins modulate several intercellular immune interactions, including adhesion, migration, synapse formation, as well as assisting in intracellular interactions as organizers of membrane-signaling complexes. They are also involved in intracellular protein transport, endocytosis, and exocytosis, and function as chaperones or stabilizers of lineage-specific molecules. CD81, a member of the tetraspanins, has been reported to be involved in an astonishing range of physiological responses [33,35,40]. Association of CD81 with various surface molecules, including MHC class II, has previously been reported. As our results demonstrated the association of CD99 with CD81 and MHC molecules, we speculate that the CD99-MHC-CD81 complex is a tetraspanin web and plays an important role in the immune response.

CD99 is expressed as two distinct isoforms, a long 32 kDa form (type I) and a short 28 kDa form (type II) resulting from an alternative splicing process of the product of the encoding gene [22]. In the immune cells, CD99 are expressed in both short and long forms [16,22]. Both isoforms have their functional roles, and differential expression can lead to distinct functional outcomes [27,30,31]. The truncation of the cytoplasmic domain of the CD99 short form may result in a loss of interaction with signaling molecules recognized by the cytoplasmic domain of the long form. It has been demonstrated that the cytoplasmic domain of the long form contains two putative phosphorylation sites, a serine at amino acid residue 168 and a threonine at amino acid residue 181. These potential phosphorylation sites may be important for intracellular signaling events and/or extracellular molecular interactions. Moreover, the S168 of CD99 long form has been reported to be a site for PKCα phosphorylation and is required for the oncosuppressor function [41]. It has also been postulated that truncation of the cytoplasmic domain of the CD99 short form causes an alteration of the three-dimensional structure, leading to different binding sites for its ligand. However, it is still unknown whether CD99 mediated signaling pathways are modulated by the differential expression of CD99 isoforms [16,22] or whether each CD99 isoform promotes different sets of signaling pathways [31]. To address the mechanisms of differing functions of the two CD99 isoforms, we investigated whether the association of CD99 with its partner molecules depended on the distinct isoform. We, however, found that both CD99 isoforms interacted with MHC and CD81 molecules. It is tempting to assume that the association of CD99 with MHC and tetraspanin CD81 may bring both CD99 isoforms into the tetraspanin web and into close proximity with the intracellular membrane signaling proteins. The molecular mechanism by which CD99 mediated signaling occurs is likely to reflect the presence of signaling molecules, such as kinases [42,43] and phosphatases [44], in the tetraspanin microdomain. This is, however, only speculation and requires further investigation.

The IS is a dynamic structure formed between T cells and antigen presenting cells, and is characterized by lipid and protein segregation, signaling compartmentalization, and bidirectional information exchange through soluble and membrane-bound transmitters [45]. The IS is the site where signals are delivered by the T cell receptors, adhesion molecules, as well as co-stimulatory and co-inhibitory receptors. The IS is divided into distinct regions: a central-supramolecular activation cluster (c-SMAC), a peripheral-(p-) SMAC, and a distal-(d-) SMAC [45]. It has been demonstrated that the c-SMAC mediates antigen recognition and subsequent T cell activation, whereas the p-SMAC supports T cell-APC conjugation and maintains the architecture of the IS. Several molecules, including MHC and tetraspanin CD81, have been shown to translocate into the IS during T cell activation [33,35,46]. Upon T cell activation, CD81 is redistributed to the contact area of T cell-APC conjugates of both T cells and APCs. Colocalization of CD81 with CD3 at the SMAC of T cells and of CD81 with MHC class II of APCs has been observed in which they function as cell surface co-stimulatory molecules. As CD99 form complexes with MHC and CD81 molecules, we investigated whether CD99 is translocated into the IS upon TCR triggering. We demonstrated that CD99 is a lipid raft-associated protein and is recruited into the IS, as has been observed for its associated molecules. Engagement of CD99 with agonistic antibody inhibits T cell activation. To our knowledge, this is the first demonstration of the accumulation of CD99 within the IS upon T cell activation and the association of CD99 with the proteins of the SMAC. We speculate that CD99-MHC-tetraspanin CD81 complexes may play an important role in T cell activation.

## Conclusions

In the present study, we demonstrate that CD99 is associated with MHC class I, MHC class II and a tetraspanin, CD81. The association was observed in both CD99 long and short isoforms. Our data collectively show that upon T cell activation, CD99 is translocated into the IS and involved in regulation of T cell proliferation. The CD99-MHC-CD81 complexes may play an important role in immune responses.

## Methods

### Antibodies, reagents and cell lines

Anti-CD99 mAbs clones MT99/3 (IgG2a) and MT99/1 (IgM) were produced in our laboratory [13,14]. Anti-CD54 mAb MEM-111 (IgG2a), anti-MHC class I mAb HC10 (IgG1), anti-MHC class II mAb MEM-136 (IgG1), anti-CD81 mAb M38 (IgG1), anti-NTAL mAb NAP-07 (IgG1), anti-Lck mAb Lck-01 (IgG1) and anti-CD45 mAb MEM-55 (IgG1) were kindly provided by Prof. Vaclav Horejsi (Institute of Molecular Genetics, Academy of Sciences of the Czech Republic, Prague, Czech Republic). Isotype matched control mAbs, PB-1 (anti-hemoglobin Bart's; IgG1) and 13 M (anti-bacteriophage protein; IgG2a) were generated in our laboratory. Isotype matched control mAbs 4G2 (anti-dengue viral protein; IgG2a) and were obtained from Dr. Prida Malasit (Division of Medical Molecular Biology, Faculty of Medicine Siriraj Hospital, Mahidol University, Bangkok, Thailand). Fluorescein isothiocyanate (FITC)- and Phycoerythrin (PE)-conjugated sheep F(ab')2 anti-mouse immunoglobulin antibodies were purchased from Chemicon Australia Pty Ltd (Victoria, Australia) and Beckman Coulter (Marseille, France), respectively. Alexa Fluor 488-labeled goat anti-mouse IgG antibodies, Alexa Fluor 568-labeled goat anti-mouse IgM antibodies, CellTrace™ Far Red DDAO-SE and Hoechst 33258 dye were obtained from Invitrogen (Carlsbad, CA, USA). Horseradish peroxidase (HRP)-conjugated rabbit anti-mouse immunoglobulin antibodies and HRP-conjugated streptavidin were obtained from Dako (Glostrup, Denmark).

Sulfo-NHS-LC-Biotin and detergent Brij-58 were obtained from Pierce (Rockford, IL, USA). Laurylmaltoside (n-dodecyl-β-D-maltoside; LM) was obtained from Calbiochem/Merck (Darmstadt, Germany). Detergent Brij-98, protease inhibitors (phenylmethylsulfonyl fluoride (PMSF), pepstatin A, aprotinin), TRITC-phalloidin and Staphylococcal enterotoxin B (SEB) were purchased from Sigma-Aldrich (St. Louis, MO, USA).

Several cell lines including Ramos (human Burkitt's lymphoma cell line), Raji (human Burkitt's lymphoma cell line), Jurkat (human T cell lymphoblast-like cell line), Molt4 (human acute lymphoblastic leukemia cell line), U937 (human leukemic monocyte lymphoma cell line) and THP-1 (human acute monocytic leukemia cell line) were used in this study. All cell lines were maintained in RPMI-1640 medium supplemented with 10% fetal calf serum (FCS) (Gibco, Grand Island, NY), 40 μg/ml gentamycin and 2.5 μg/ml amphotericin B in a humidified atmosphere of 5%CO_2 _incubator at 37°C.

### Co-immunoprecipitation of cell surface molecules

Jurkat cells were labeled with 5 mM Sulfo-NHS-LC-Biotin at 4°C for 1 hr. The biotinylation was quenched by washing once with 1 mM glycine in PBS and twice with PBS. The biotinylated cells (5 × 10^7 ^cells) were solubilized for 30 min on ice in 1 ml lysis buffer (20 mM Tris-HCl pH 7.5, 100 mM NaCl, 2 mM EDTA, 50 mM NaF, 1 mM Na_3_VO_4_, 5 mM iodoacetamide, and protease inhibitors) containing either detergent 1% Brij-58 or 1% LM. The cell suspension was clarified by centrifugation at 10,000 × g for 30 min at 4°C. The clarified cell lysates were precleared with protein G Sepharose beads (Pierce) coated with mouse immunoglobulins. The precleared lysates were then incubated with 10 μg of purified mAbs for 2 hr at 4°C. Protein G Sepharose beads were added and the mixtures were rotated overnight at 4°C. After incubation, beads were washed five times, and proteins were dissociated from the Protein G beads by addition of SDS-reducing sample buffer (62.5 mM Tris-HCl pH 6.8, 5% β-mercaptoethanol, 2% sodium dodecyl sulfate (SDS), 10% glycerol, and 0.01% bromophenol blue) and boiled for 5 min. The precipitated proteins were then resolved by SDS-polyacrylamide gel electrophoresis (PAGE) and subsequently transferred to a nitrocellulose membrane (Pall Corp., East Hill, NY, USA). The membrane was blocked overnight in PBS containing 5% BSA at 4°C. The blocked membrane was incubated for 1 hr at room temperature with HRP-conjugated streptavidin. The reactive protein bands were visualized by the chemiluminescence detection system (Pierce).

### Co-immunoprecipitation of un-labeled cell lysates and Western immunoblotting

The tested cell lines (5 × 10^7 ^cells) were pelleted and lysed with 1 ml lysis buffers containing either 1% Brij-58 or 1% LM for 30 min on ice. The lysates were clarified by centrifugation. The immunoprecipitations were performed according to the method described above. The precipitated proteins were resolved by SDS-PAGE under non-reducing conditions and transferred to a nitrocellulose membrane, followed by Western immunoblotting experiments. The nitrocellulose membranes were blocked with 5% BSA in PBS at room temperature for 1 hr. Then, the blocked membrane was incubated with mAbs for 1 hr at room temperature. After being washed five times with 0.1% Tween-20 in PBS, the membrane was incubated with HRP conjugated rabbit anti-mouse immunoglobulin antibodies for 1 hr. The specific protein bands were then visualized by the chemiluminescence detection system (Pierce).

### Generation of CD99 expressing and CD99 non-expressing Ramos cells by immunomagnetic cell sorting

Ramos cells were stained with anti-CD99 mAb MT99/3 for 30 min on ice. Then, FITC-conjugated anti-mouse immunoglobulin antibodies were added and incubated for 30 min on ice. After incubation, cells were washed and incubated with anti-FITC MicroBeads (Miltenyi Biotec, Bergisch Gladbach, Germany) according to the manufacturer's instructions. Cells were washed and resuspended in MACS sorting buffer (0.5% BSA, 2 mM EDTA in PBS) and sorted with an AutoMACS cell sorter (Miltenyi Biotec). The obtained positive and negative cell fractions were collected and cultured in RPMI-1640 medium supplemented with 10% fetal bovine serum (FBS) (Gibco, Grand Island, NY, USA), 40 mg/ml gentamicin and 2.5 mg/ml amphotericin B in a humidified atmosphere of 5% CO_2 _at 37°C. CD99 expression on the sorted cells was verified by flow cytometric analysis.

### Co-localization analysis

Peripheral blood mononuclear cells (PBMCs) were isolated from heparinized whole blood of healthy donors by Ficoll-Hypague density gradient centrifugation. PBMCs or Raji B cell line (1 × 10^7 ^cells/ml) was pre-incubated with 10% human serum (blood group AB) at 4°C for 30 min. For staining of the surface membrane proteins, 50 μl of the cell suspension were incubated with an equal volume of 20 μg/ml anti-MHC class I, anti-MHC class II, anti-CD81 mAbs, or isotype matched control PB-1 mAb (for Raji) or MEM-111 mAb (for PBMCs) at 4°C for 30 min. The cells were washed twice with PBS containing 1%BSA and 0.02% NaN_3 _(1% BSA-PBS-NaN_3_) and then incubated with Alexa Fluor 488-conjugated goat F(ab')_2 _anti-mouse IgG antibodies at 4°C for 30 min. After twice washing, the cells were then incubated with mouse immunoglobulins at 4°C for 30 min to neutralize the reactivity of the adding conjugates. Subsequently, CD99 molecules were stained using anti-CD99 mAb MT99/1 (IgM isotype) at 4°C for 30 min, followed by Alexa Fluor 568-conjugated goat F(ab')_2 _anti-mouse IgM antibodies at 4°C for 30 min. Finally, cells were fixed with 4% paraformaldehyde for 15 min at room temperature and plated on poly-D-lysine coated slides. Cell nuclei were visualized with Hoechst 33258 dye. For evaluation of colocalization, cells were visualized and images were acquired using a confocal laser scanning microscope (LSM 700; Zeiss, Le Pecq, France).

### Generation of Romos cell expressing CD99 short and long isoforms

Plasmid DNA encoding full-length CD99 cDNA [13] was used as a template for generation of cDNA encoding CD99 short and long forms by the polymerase chain reaction (PCR). The PCR products were inserted into the *EcoR*I site of the MSCV retroviral vectors, according to the manufacturer's instructions (Clonetech Laboratory, Mountain View, CA, USA). The obtained plasmid DNA were transformed into *E. coli *TOP10 (Invitrogen). The plasmid DNA were then isolated from the transformed *E*. *coli *by Qiagen chromatography columns (Qiagen, Hilden, Germany). The inserted genes in the constructed plasmids were checked by restriction fragment analysis using corresponding restriction enzymes and DNA sequencing.

To prepare retroviruses (RV) harboring plasmid carrying CD99 isoforms, the plasmid DNA were transfected into Phoenix-Ampho cells (Origene, Rockville, MD, USA) using Lipofectamine (Invitrogen). At 48 hr post-transfection, RV-containing supernatants were collected and clarified by centrifugation. The RV were then used to spin-infect (1200 × g, 90 min at 32°C) the Ramos CD99 negative cells in the presence of polybrene (10 μg/ml)(Sigma-Aldrich). Cells were allowed to expand in culture and sorted by immunomagnetic cell sorting to isolate the CD99 expressing cells. Stable transfectant clones with high CD99 expression were identified by flow cytometric analysis.

### Flow cytometric analysis

Cells (1 × 10^7^cells/ml) were pre-incubated with 10% human serum (blood group AB) at 4°C for 30 min to block nonspecific Fc-receptor-mediated binding of mAbs. For staining of the surface membrane proteins, 50 μl of the cell suspension were incubated with an equal volume of 20 μg/ml anti-CD99 mAb MT99/3 at 4°C for 30 min. The cells were washed twice with 1% BSA-PBS-NaN_3 _and then incubated with PE-conjugated sheep F(ab')_2 _anti-mouse immunoglobulin antibodies at 4°C for 30 min. Cells were washed and membrane fluorescence was analyzed by a flow cytometer (FACSCalibur; Becton Dickinson, Sunnyvale, CA, USA).

### Immunological synapse and confocal image analysis

Raji B cells (1 × 10^7 ^cells/ml) were loaded with 1 μg/ml of staphylococcal enterotoxin B (SEB) (Sigma-Aldrich) for 15 min at 37°C. Jurkat T cells (1 × 10^7 ^cells/ml) were labeled with CellTrace™ Far Red DDAO-SE. After washing, an equal number of Jurkat cells were mixed with Raji cells and incubated at 37°C for 15 min. Cell mixtures were placed on poly-D-lysine coated slides, fixed with 4% paraformaldehyde for 15 min at room temperature and permeabilized with 0.1% Triton X-100 for 5 min on ice. Cells were then incubated with anti-CD99 mAb MT99/3 for 30 min at room temperature. Alexa Fluor 488-labeled goat anti-mouse IgG secondary antibody and TRITC-phalloidin were added and incubated for 30 min. Cell nuclei were stained with Hoechst 33258 dye. The stained cells were analyzed and images were acquired using a confocal laser scanning microscope (LSM 700; Zeiss, Le Pecq, France).

### Determination of CD99 in lipid rafts

Lipid rafts were isolated according to the method described elsewhere [47]. Briefly, cells (1 × 10^8^) were resuspended in 0.4 ml of ice-cold hypotonic buffer (10 mM HEPES pH 7.4, 42 mM KCl, 5 mM MgCl_2_, and protease inhibitors), incubated on ice for 15 min and then passed 10 times through the 30-gauge needle. The suspension was centrifuged for 5 min, 300 × g, at 2°C to remove nuclei. The supernatant was re-centrifuged for 10 min, 25,000 × g, at 2°C to sediment the membranes. Membranes were then lysed in 0.2 ml of lysis buffer containing 1% Brij-98 or 1% LM for 30 min on ice, and spun at 10,000 × g for 30 min. The clarified lysates (0.1 ml) were applied at the top of a 1 ml Sepharose 4B column (Sigma-Aldrich) and sequentially washed with 0.1 ml of the lysis buffer. The fractions (0.1 ml) were collected at 4°C and analyzed for the presence of CD99 by SDS-PAGE and Western immunoblotting.

### Proliferation assay

PBMCs were isolated from heparinized whole blood of healthy donors by Ficoll-Hypague density gradient centrifugation. Cells (1 × 10^7 ^cells/ml in PBS) were incubated with 0.5 μM carboxyfluorescein diacetate succinimidyl ester (CFSE; Molecular Probes, Eugene, OR, USA) at 37°C for 10 min. The CFSE staining was terminated by adding cold RPMI-1640 medium supplemented with 10% fetal bovine serum (FBS; Gibco, Grand Island, NY, USA), 40 mg/ml gentamicin, and 2.5 mg/ml amphotericin B (10% FBS-RPMI 1640). The cells were then washed twice and resuspended with 10% FBS-RPMI 1640. To determine the influence of anti-CD99 mAb on T cell activation, CFSE labelled PBMCs (5 × 10^5 ^cells) were cultured in 96-well plate with or without immobilized anti-CD3 mAb OKT3 (60 ng/ml) in the presence or absence of 20 μg/ml anti-CD99 mAb or isotype matched control mAb. The plate was cultured at 37°C in a 5% CO_2 _incubator for 5 days. Cells were then harvested and determined for cell proliferation by flow cytometry (Becton Dickinson).

## Competing interests

The authors declare that they have no competing interests.

## Authors' contributions

SP conceived of the study and participated in design, carried out the immunoprecipitation analysis and drafted the manuscript. PO participated in the design of the study and performed the cell sorting and generation of stable cell lines. TB participated in the design of the study and was involved in drafting the manuscript. WL carried out the colocalization analysis. KM carried out the proliferation assay. WK is principal investigator, conceived the study and designed the experimental strategies and finalized the manuscript. All authors have read and approved the final manuscript.
